# Comparative Genomic Analysis of Seven *Vibrio alginolyticus* Strains Isolated From Shrimp Larviculture Water With Emphasis on Chitin Utilization

**DOI:** 10.3389/fmicb.2022.925747

**Published:** 2022-07-26

**Authors:** Ming Xue, Xuemin Huang, Jiawei Xue, Runduan He, Guojian Liang, Huafang Liang, Jianyong Liu, Chongqing Wen

**Affiliations:** Fisheries College, Guangdong Ocean University, Zhanjiang, China

**Keywords:** *Vibrio alginolyticus*, pan-genome, mobile genetic element, chitin utilization, shrimp larviculture

## Abstract

The opportunistic pathogen *Vibrio alginolyticus* is gaining attention because of its disease-causing risks to aquatic animals and humans. In this study, seven *Vibrio* strains isolated from different shrimp hatcheries in Southeast China were subjected to genome sequencing and subsequent comparative analysis to explore their intricate relationships with shrimp aquaculture. The seven isolates had an average nucleotide identity of ≥ 98.3% with other known *V. alginolyticus* strains. The species *V. alginolyticus* had an open pan-genome, with the addition of ≥ 161 novel genes following each new genome for seven isolates and 14 publicly available *V. alginolyticus* strains. The percentages of core genes of the seven strains were up to 83.1–87.5%, indicating highly conserved functions, such as chitin utilization. Further, a total of 14 core genes involved in the chitin degradation pathway were detected on the seven genomes with a single copy, 12 of which had undergone significant purifying selection (*dN*/*dS* < 1). Moreover, the seven strains could utilize chitin as the sole carbon-nitrogen source. In contrast, mobile genetic elements (MGEs) were identified in seven strains, including plasmids, prophages, and genomic islands, which mainly encoded accessory genes annotated as hypothetical proteins. The infection experiment showed that four of the seven strains might be pathogenic because the survival rates of *Litopenaeus vannamei* postlarvae were significantly reduced (*P* < 0.05) when compared to the control. However, no obvious correlation was noted between the number of putative virulence factors and toxic effects of the seven strains. Collectively, the persistence of *V. alginolyticus* in various aquatic environments may be attributed to its high genomic plasticity via the acquisition of novel genes by various MGEs. In view of the strong capability of chitin utilization by diverse vibrios, the timely removal of massive chitin-rich materials thoroughly in shrimp culture systems may be a key strategy to inhibit proliferation of vibrios and subsequent infection of shrimp. In addition, transcontinental transfer of potentially pathogenic *V. alginolyticus* strains should receive great attention to avoid vibriosis.

## Introduction

Members of the genus *Vibrio*, are ubiquitously distributed in aquatic environments, and thus have particularly intricate relationships with aquatic organisms (Takemura et al., 2014; Le Roux and Blokesch, 2018). On the one hand, vibrios are considered a part of the normal microbiota of aqueous environments; planktonic and particle-associated vibrios seem to enhance the survival and growth of aquaculture species (Gomez-Gil et al., 1998; Xue et al., 2016; Kumar et al., 2017). On the other hand, vibrios are frequently reported as the major opportunistic pathogens causing vibriosis in cultured aquatic animals (Ruwandeepika et al., 2012; Xue et al., 2020; de Souza Valente and Wan, 2021). In general, most bacterial members of the Harveyi clade, such as *V*. *parahaemolyticus* and *V. harveyi*, have long been implicated in vibriosis in shrimp aquaculture, e.g., *V. parahaemolyticus* is the causative agent of acute hepatopancreatic necrosis disease (AHPND) (Han et al., 2020), whereas *V. harveyi* is closely associated wtih luminescent vibriosis (Vandenberghe et al., 1999; Ruwandeepika et al., 2010; Wang et al., 2015). In contrast, less attention has been paid to *V. alginolyticus*, although this opportunistic aquatic pathogen is increasingly associated with outbreak of shrimp vibriosis **(**Liu et al., 2004; Ren et al., 2013; Abdul Hannan et al., 2019; Bachand et al., 2020; de Souza Valente and Wan, 2021**)**. Furthermore, some *Vibrio* species, including *V. alginolyticus*, are human pathogens that cause severe gastroenteritis and extra-intestinal diseases (Austin, 2010; Janda et al., 2015; Jacobs Slifka et al., 2017). Collectively, various vibrios can be pathogenic, non-pathogenic, or even beneficial, depending on the heterogeneity of diverse intraspecies strains in terms of pathogenicity, virulence, and antibiotic resistance (Thompson et al., 2009, 2010; Lajnef et al., 2012; Busschaert et al., 2015; Sun et al., 2021).

In addition to their potential pathogenicity, vibrios play an important role in nutrient cycling in aquatic environments by hydrolyzing polysaccharides and taking up dissolved organic matter (Zhang et al., 2018). For instance, the ubiquity of vibrios is speculated to be due to their ability to degrade chitin (Hunt et al., 2008; Lin et al., 2018). Hunt et al. (2008) proposed a chitin degradation pathway by comparing 19 *Vibrio* and *Photobacterium* genomes. Markov et al. (2015) also suggested that chitin degradation by *V. cholerae* is a catabolic cascade completed by a combination of multiple chitinases, chitin-binding proteins, and related regulators, which play special roles in *V. cholerae* ecology, such as chemotaxis and biofilm formation. Among the genes responsible for chitin utilization, *chi*A (chitinase gene A) has the highest expression in response to crab shell chitin (Meibom et al., 2004) and may be a potential indicator of chitinoclastic ability because it is highly conserved in vibrios (Lin et al., 2018). Although some genes encoding chitinases of vibrios have been reported, genetic variation of chitin degradation-related genes involved in a complete pathway remains unclear.

With the advent of ultra-rapid genome sequencing, pan-genome and comparative genomic analyses have attracted considerable attention because of their accurate and comprehensive results (Rasko et al., 2008; Tettelin et al., 2008; Zhao et al., 2012; Seemann, 2014; Page et al., 2015). For example, by integrating a variety of *Vibrio* genomes, Lin et al. (2018) reported that this genus encompasses a steady core genome and a tremendous pan-genome with substantial gene gain in evolutionary history. A variety of mobile genetic elements (MGEs), identified by genomic analysis, have been considered to facilitate the evolution and niche adaptation of vibrios via horizontal gene transfer (HGT) (Thompson et al., 2009; Hazen et al., 2010; Le Roux and Blokesch, 2018). The similarity of the hemolysin gene of *V. alginolyticus* to virulence factor *trh* (thermostable direct hemolysin (*tdh*)-related hemolysin) of *V. parahaemolyticus* may indicate the occurrence of HGT (González-Escalona et al., 2006). Hehemann et al. (2016) also reported the acquisition of alginate-degradation genes in *V. breoganii* via HGT, which initiates metabolic pathway diversification. Similarly, Deng et al. (2019) suggested that the exchange of virulence factors and resistance genes via HGT among 31 *V. harveyi* strains contributes to pathogenicity and drug resistance. Intrinsically, phenotypic differences in various *Vibrio* species or strains are all driven by the acquisition of unique genes via diverse MGEs (Hastings et al., 2004; Le Roux and Blokesch, 2018). To date, the number of *V. alginolyticus* genomes uploaded in the NCBI database is continuously increasing; however, approximately 85% of which are draft genomes. Zheng et al. (2021) reported the population composition, virulence distribution, and antibiotic resistance factors of *V. alginolyticus* using draft genomes. An analysis with complete genomes of *V. alginolyticus* was conducted by Chibani et al. (2020), who found that genomic variation among nine closely related *V. alginolyticus* strains is mainly located on MGEs and speculated that these strains may be derived from a habitat-specific ecotype through clonal expansion.

As well known, chitin and its derivatives are important nutrients for penaeid shrimp, but the digestibility on chitin by shrimps themselves is relatively low (Clark et al., 1993; Shiau and Yu, 1998). Given the high efficiency of vibrios in chitin-degradation and wide distribution of vibrios in shrimp rearing environments, an in-depth knowledge of genomic contexts of vibrios could help to treat them differentially, i.e., neutral or beneficial vibrios could be applied to facilitate chitin digestion and absorption in intestine to improve shrimp growth, meanwhile, the risks of vibriosis outbreak should be controlled due to the proliferation of potentially pathogenic vibrios after utilizing chitin or chitin-rich shells/exoskeletons which are prevalent in shrimp ponds. In the present study, the genomes of seven *V. alginolyticus* strains isolated from shrimp larviculture ponds across four provinces in Southeast China were extensively characterized using a detailed pan-genome analysis, then the toxicity of the seven isolates to shrimp postlarvae and their chitin utilization were also determined. Through a comprehensive analysis of phylogeny and genetic contents of different *V. alginolyticus* strains, together with their pathogenicity and chitinolytic ability, these results may contribute to growth and prevention of vibriosis of penaeid shrimp.

## Materials and Methods

### Strains and Media

Seven *Vibrio* strains analyzed in this study were obtained from August 2015 to May 2019, which were isolated from rearing water of different hatcheries of the Pacific shrimp (*Litopenaeus vannamei*) located in four provinces in Southeast China. For each hatchery, 3–10 vibrios isolates were obtained and one representative of *V. alginolyticus* was selected after their 16S rRNA genes were primarily sequenced (data not shown). All strains were stored as frozen cultures in 20% glycerol at −80°C. Details on strain designation, the sampling site and isolation time are listed in Table 1. The 2216E agar (HB0132; Hopebio, China) was used for routine culture. Colloidal chitin agar medium (pH 7.0–7.2), which consisted of 0.7 g K_2_HPO_4_, 0.3 g KH_2_PO_4_, 0.5 g MgSO_4_, 0.02 g FeSO_4_·H_2_O, 20 g sea salt, 20 g agar, and 5 g colloidal chitin (2%, w/v) per liter, was used to examine the chitinolytic capability of the strains.

**Table 1:** General features of the seven *V. alginolyticus* strains in this study.

**Strain designation**	**Isolation site and time**	**Chromosome/ Plasmid**	**Size (bp)**	**G+C (%)**	**GenBank accession no**.	**No. of CDS**	**No. of tRNA**	**No. of rRNA**	**No. of nc RNA**
XWV9	Zhanjiang, Guangdong; 2016-07	ChrI	3,352,833	44.7	CP082319	3,016	116	34	21
		ChrII	1,885,978	44.5	CP082320	1,638	13	3	8
		pL93	93,384	46.2	CP082321	114	0	0	1
HYV1	Zhanjiang, Guangdong; 2019-05	ChrI	3,342,267	44.7	CP082310	2,924	116	34	19
		ChrII	1,826,343	44.6	CP082311	1,633	13	3	8
		pL40 (linear)	40,196	39.7	CP082312	49	0	0	0
ZLV3	Zhanjiang, Guangdong; 2015-08	ChrI	3,294,198	44.8	CP082315	2,935	116	34	19
		ChrII	1,826,965	44.6	CP082316	1,596	13	3	8
SXV3	Zhanjiang, Guangdong; 2017-04	ChrI	3,396,607	44.8	CP082317	3,065	116	34	21
		ChrII	1,846,558	44.7	CP082318	1,607	13	3	8
FJV2	Zhangzhou, Fujian; 2016-08	ChrI	3,379,103	44.6	CP082303	3,025	116	34	19
		ChrII	1,813,378	44.6	CP082304	1,594	13	3	8
		pL33_1	33,998	42.8	CP082305	41	0	0	0
		pL33_2	33,975	42.8	CP082306	45	0	0	0
HNV2	Wenchang, Hainan; 2016-07	ChrI	3,266,502	44.8	CP082307	2,892	116	34	19
		ChrII	1,843,725	44.6	CP082308	1,612	14	3	8
		pL90	90,854	46.1	CP082309	109	0	0	0
ZZV2	Beihai, Guangxi; 2017-03	ChrI	3,329,458	44.7	CP082313	3,008	116	34	19
		ChrII	1872924	44.5	CP082314	1,638	13	3	9

### DNA Extraction and Genome Sequencing

The genomic DNA from the overnight cultures of the seven strains in 2216E liquid medium was extracted using the QIAamp DNA Mini Kit (Cat#51304; QIAGEN), according to the manufacturer's instructions. The DNA purity (OD 260/280: 1.8–2.0, and OD 260/230: 2.0–2.2) and quantity were detected using the NanoDrop™ One UV-Vis spectrophotometer (Thermo Fisher Scientific, USA) and the Qubit^®^ 3.0 Fluorometer (Invitrogen, USA), respectively. For each strain, a 1D template library was constructed and sequenced on the Oxford Nanopore sequencing platform GridION (strains FJV2, SXV3, XWV9, ZLV3, and ZZV2) and platform PromethION (HNV2 and HYV1) (Oxford Nanopore, Oxford, UK) at NextOmics Biosciences Co., Ltd, Wuhan, China. The long sequences of FJV2, SXV3, XWV9, ZLV3, and ZZV2 were assembled using Canu v1.7.11 (Koren et al., 2017), and those of HNV2 and HYV1 were assembled using Fyle v2.6 (Kolmogorov et al., 2019). For each strain, the assembled contigs were subjected to a built-in plasmid database with data downloaded from NCBI (https://ftp.ncbi.nlm.nih.gov/refseq/release/plasmid/), plasmid was identified when the alignment length was >20% of the total sequence length (<1 Mb). The complete genome sequences of the seven strains were deposited in GenBank with accession numbers listed in Table 1.

Available complete genomes of other 14 *V. alginolyticus* strains were downloaded from NCBI (ftp://ftp.ncbi.nlm.nih.gov/genomes/). Basic information about the replicon, size, number of coding sequence (CDS), and accession number of these strains were described by Chibani et al. (2020). Among them, strain ATCC 17749 was isolated from spoiled horse mackerel (*Trachurus trachurus*) that causes food poisoning in Japan (Liu et al., 2015). Strain ATCC 33787 was isolated from seawater in Oahu, Hawaii, USA (Wang et al., 2016). Strain ZJ-T was isolated from diseased *Epinephelus coioides* in Zhanjiang, Guangdong Province, China (Chang et al., 2009). Meanwhile, the information on strains FDAARGOS_108, FDAARGOS_110, and FDAARGOS_114, which were isolated in England, were obtained from the Food and Drug Administration, USA. The remaining eight strains (K01M1, K04M1, K04M3, K04M5, K05K4, K06K5, K08M3, and K10K4) were isolated from pipefish (*Syngnathus typhle*) in Germany (Chibani et al., 2020).

### Calculation of Average Nucleotide Identity (ANI)

The pairwise ANI indices among the seven strains and the 21 strains were calculated using FastANI (https://github.com/ParBLiSS/FastANI) (Jain et al., 2018). The resulting matrix was clustered and visualized in R v4.1.0 using the package pheatmap (https://cran.r-project.org/web/packages/pheatmap/index.html). The complete genomes of *V. parahaemolyticus* LVP66, *V. parahaemolyticus* AM51552, *V. harveyi* QT520, and *V. harveyi* ATCC 33843 were downloaded from the NCBI database.

### Genome Annotation

Gene prediction and functional annotation were performed using Prokka v1.13 with default settings (Seemann, 2014). This program uses a rapid hierarchical approach to classify proteins using databases derived from UniProtKB (https://www.uniprot.org/help/uniprotkb). Genomic islands (GIs) were identified using IslandViewer 4 (http://www.pathogenomics.sfu.ca/islandviewer/) (Dhillon et al., 2013). Prophages were predicted using the phage search tool (PHAST, http://phaster.ca/), wherein only intact prophages (score > 90) were retained, and questionable (70–90) and incomplete (< 70) prophages were discarded (Arndt et al., 2016). The presence of clustered regularly interspaced short palindromic repeats (CRISPR) was examined using CRISPRFinder (http://crispr.u-psud.fr/Server/CRISPRfinder.php).

### Identification of Virulence and Antibiotic Resistance Genes

Putative virulence factors of the seven strains were predicted by aligning against the Virulence Factor Database (VFDB) (http://www.mgc.ac.cn/VFs/) (Liu et al., 2019). Acquired antibiotic resistance genes (ARGs) were blasted with CARD-rgi against the Antibiotic Resistance Genes Database (ARDB) (http://ardb.cbcb.umd.edu/) (Liu and Pop, 2009) and further identified using ResFinder 4.1 (https://cge.cbs.dtu.dk//services/ResFinder/). In both ARDB and Resfinder, only hits showing ≥ 95% identity and ≥ 60% length coverage were considered as ARGs.

### Pan-Genome Analysis

Following genome annotation using Prokka, the annotation files and the functional information of Cluster of Orthologous Groups (COG) of the seven isolates, together with those of the other 14 *V. alginolyticus* strains, were subjected to the pan-genome pipeline of PGAP, as described by Zhao et al. (2012). This pipeline applies the Markov cluster algorithm (http://micans.org/mcl/) to perform homologous gene clustering, where whole genes denote genes in all strains, core genes represent orthologous genes shared by all strains, dispensable and specific genes denote those that are not included in at least one of the strains and in only one strain, respectively. The Phylip software (Retief, 2000) was used to construct a phylogenetic tree based on pan-genome with method of UPGMA (unweighted pair-group method with arithmetic means). The COG functional categories regarding whole, core, dispensable, and specific genes of the strains were also determined using the PGAP module.

The core genome alignment of the 21 *V. alginolyticus* strains was obtained with standard settings (minimum BLASTP identity of 90%) after the gff3 files, derived from Prokka annotation, were subjected to Roary v3.8.0 (Page et al., 2015). The phylogenetic tree was constructed based on the concatenation of 3894 single-copy core genes with RAxMLv8.1.22, using the maximum-likelihood algorithm (Stamatakis, 2014). To describe the pan-genome, a model of y=Ax^B^+C was adopted to fit the pan-genome curve, and y=Ae^Bx^+C and y=Ax^B^ were used to fit the core genome and new gene curves, respectively, y is the number of the respective whole genes/core genes/new genes, x is the number of genomes, and A, B, and C are the corresponding constants of the fitted curves. The exponent B in the pan-genome curve is an indicator of whether the pan-genome is open (B < 1) or closed (B > 1) (Tettelin et al., 2008), all of which were visualized using PanGP v1.0.1 (Zhao et al., 2014). The MATLAB R2021a software (https://ww2.mathworks.cn/) was used to demonstrate the pan-genome profile with the binary pan-genome matrix of the presence or absene of genes in 21 strains resulting from Roary as input data. Likewise, to show the relationship between specific or shared gene clusters among the seven strains, the binary matrix file of the seven strains was used as the input data in R v4.1.0, using the package UpSetR (https://cran.r-project.org/web/packages/UpSetR/).

### Analysis of Genes Related With Chitin-Utilization

Using the functionally annotated files derived from Prokka, a total of 14 core genes involved in chitin utilization were identified according to the chitin degradation pathway defined for *V. cholerae* (Hunt et al., 2008; Markov et al., 2015). To explore the phylogenetic relationships of the seven strains based on these genes, a tree was generated based on concatenation of 14 gene sequences using the neighbor-joining method implemented in MEGA X (Kumar et al., 2018), then the newick format file was subjected to the package genoPlotR (http://genoplotr.r-forge.r-project.org/) in R v4.1.0 to produce a composite graph using the function plot_gene_map. For the seven strains, varying information about the genes, including insertion-deletion (indel), non-synonymous mutation, and synonymous mutation, were analyzed using the CDS variation module of the PGAP pipeline.

### Infection of Shrimp Postlarvae With the Seven Strains

To evaluate the potential toxicity of the seven strains to shrimp, 32 food grade polypropylene barrels (25 l) with 10 l disinfected seawater were randomly divided into eight groups with four replicates. The seawater (salinity 27.5‰, pH 7.9, 28 ± 1°C) was aerated constantly after sand filtration. After acclimatization for 2 d, each barrel was randomly supplied with 280 healthy shrimp postlarvae at stage 8 (~7 mm of body length), which were obtained from the specific pathogen-free broodstock in a commercial hatchery. At the initial and at 24 h, the rearing water of seven groups was treated with the logarithmic-phase cells of seven strains at concentration of 2 × 10^6^ CFU ml^−1^, respectively, while one group left randomly used as the control with no addition of bacterial cells. The postlarvae were fed diet of shrimp crackers four times a day, and the surviving individuals were determined after 3 days of infection.

### Statistical Analysis

Shrimp survival rates (%) are represented as the mean ± SD. After completion of arcsine square root conversion, the survival rates were subjected to one-way analysis of variance (ANOVA) to determine the significance (*P* < 0.05), followed by Tukey's HSD *post hoc* test when a significant difference was detected using the package agricolae in R v4.1.0.

## Results

### General Features of the Seven *V. alginolyticus* Strains

Basic information on the seven isolates and their genomes are presented in Table 1. Each of the seven genomes contained a 3.27–3.40 Mb large chromosome I and a 1.81–1.89 Mb small chromosome II. The G+C content of the seven strains ranged in 44.5–44.8%, which is common for *Vibrio* species. Four of the seven isolates had one or two extra-chromosomal plasmids (34.0–93.4 kb), with G+C contents ranging from 39.7 to 46.2%. Overall, a genome size of 4,843 ± 77 genes was predicted for the seven *V. alginolyticus* strains.

### ANI Analysis

**Figure 1:**
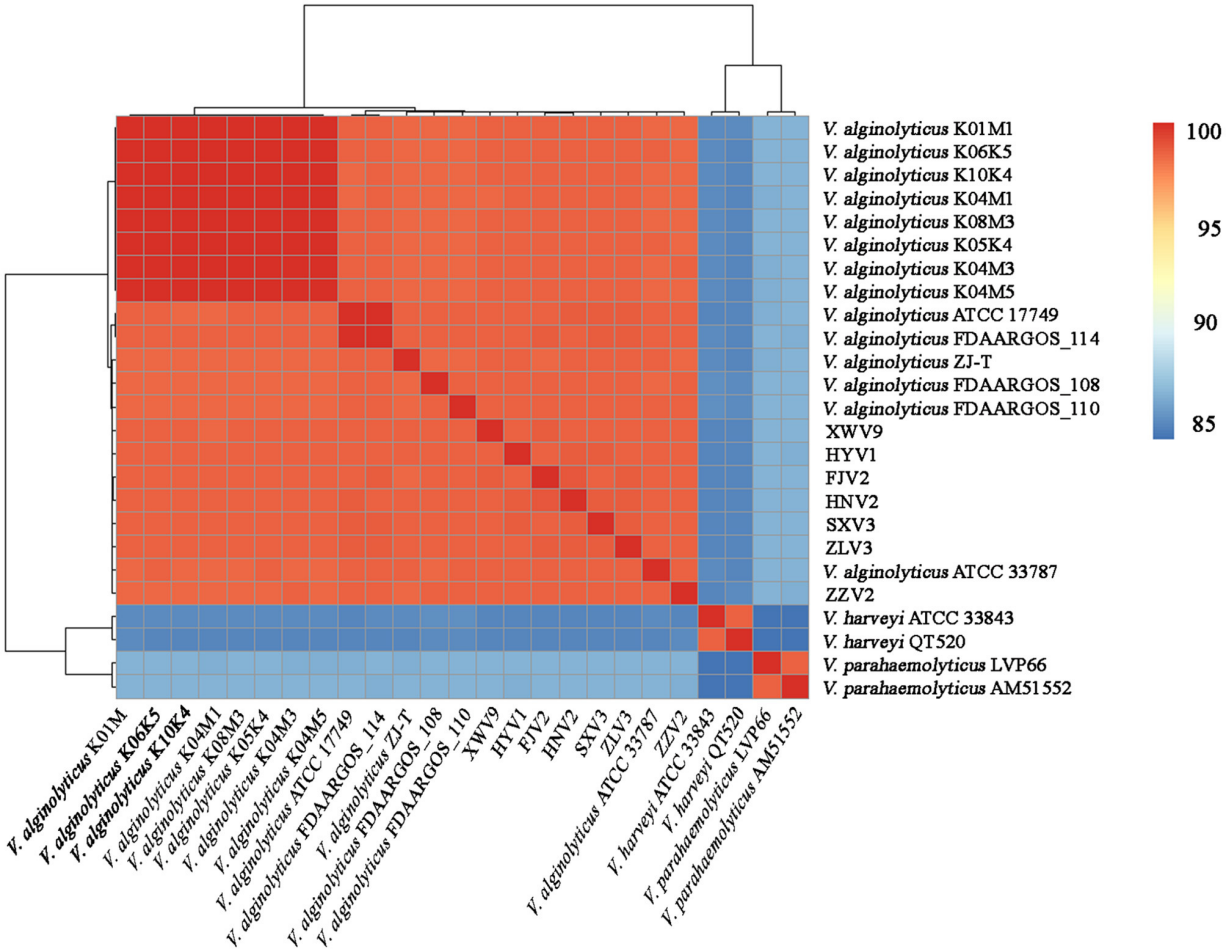
Average nucleotide identity analysis of the complete genomes of 21 *V. alginolyticus* strains, together with *V. harveyi* ATCC 33843 and QT520, and *V. parahaemolyticus* LVP66 and AM51552.

The seven strains shared > 98.4% ANIs and also shared high ANIs (≥ 98.3%) with other known *V. alginolyticus* strains, suggesting that these isolates all belonged to *V. alginolyticus*. Meanwhile, the seven strains had ANIs of 84.6–84.9% and 83.9–86.3% with closely related species of *V. harveyi* and *V. parahaemolyticus*, respectively (Figure 1), which were below the species threshold of 95–96% ANI.

### Pan-Genome Analysis

**Figure 2:**
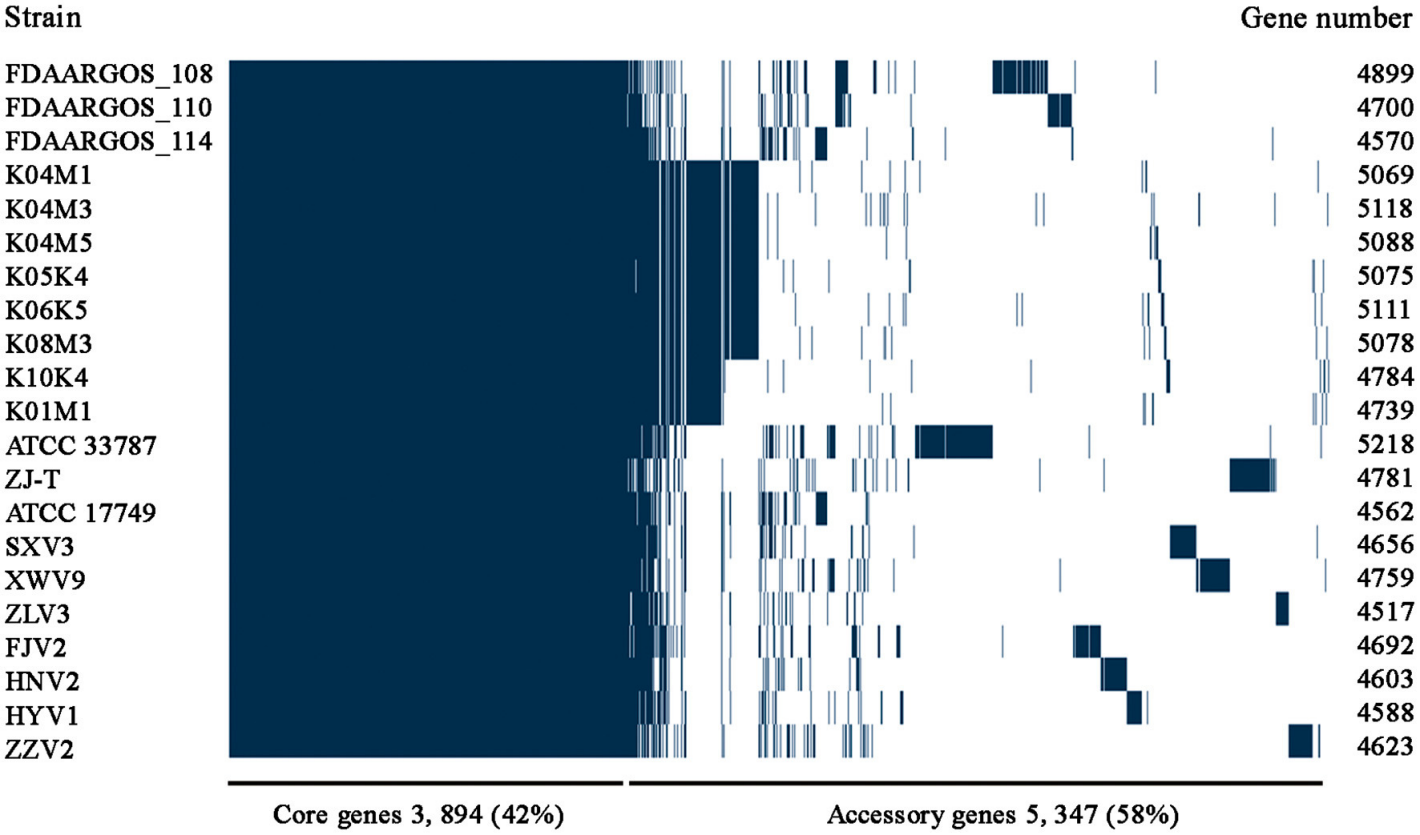
The pan-genome profiles of the seven *V. alginolyticus* strains isolated in this study, together with other 14 available genomes of *V. alginolyticus* strains.

**Figure 3:**
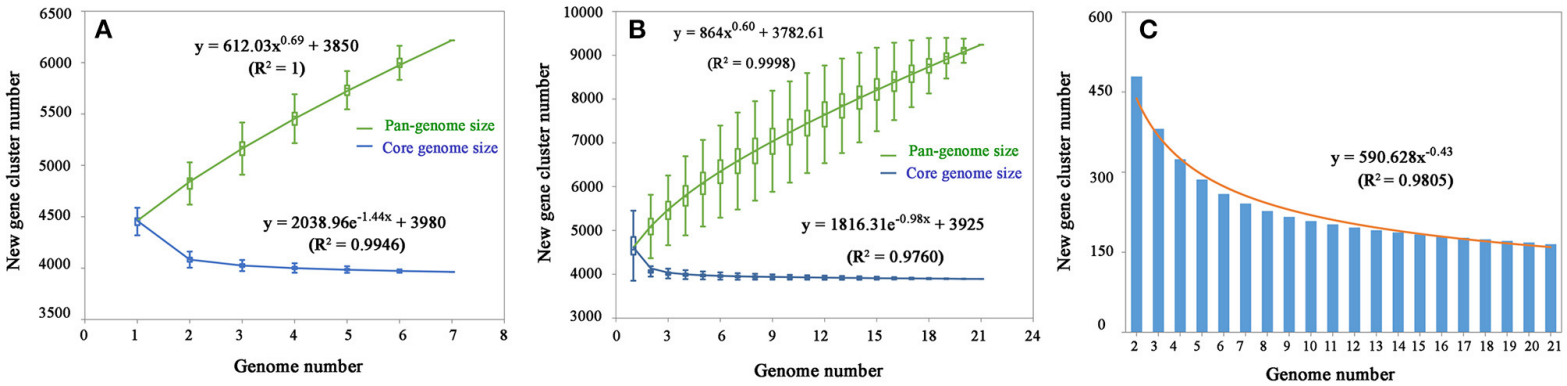
The profiles of pan-genome and core genes of the seven strains **(A)** and 21 strains **(B)** of *V. alginolyticus*, and the profile of increased new genes in the 21 strains **(C)**.

After analysis with the PGAP pipeline, 6,217 gene clusters were detected in the pan-genome of the seven strains, while the gene clusters increased to 9,241 when 21 strains were considered (Figure 2). Accordingly, 3,963 and 3,894 shared genes constituted the core genomes of the seven and 21 *V. alginolyticus* strains, respectively. The data of whole and core genes were fitted well by a power function and an exponential decay function, respectively (Figures 3A,B), and the repertoire of new genes was also fitted by an exponential decay function (Figure 3C). Notably, at least 256 and 161 new genes were identified for each genome in the seven and 21 strains, respectively. Moreover, the exponent B (~ 0.60) in the pan-genome formula was lower than 1.0, indicating that *V. alginolyticus* had an open pan-genome.

**Figure 4:**
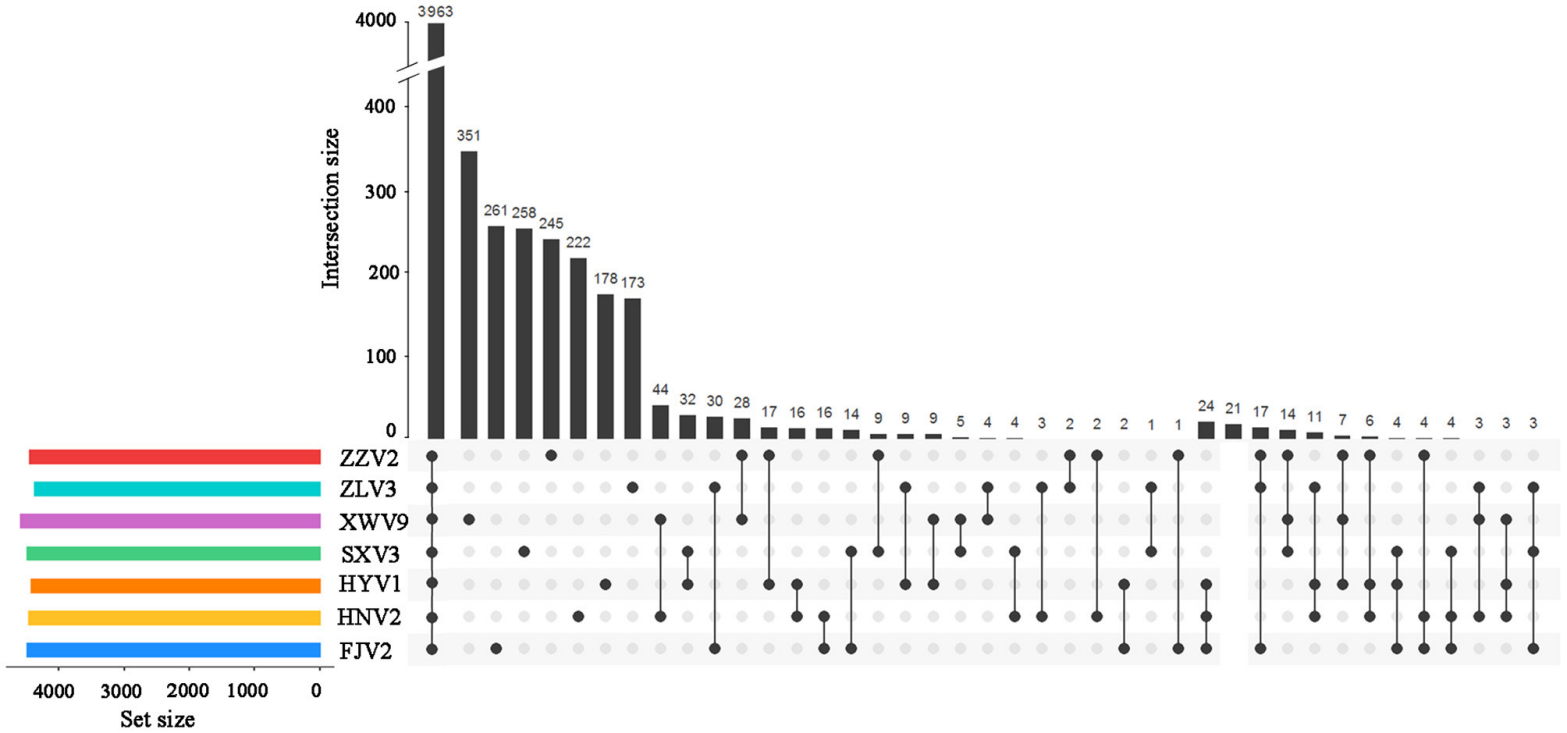
UpSet plot of the intersection of gene clusters in the seven *V. alginolyticus* strains. The numbers of gene clusters in the core, specific, and intersecting genomes in two or three strains are indicated.

Among the 6,217 genes of pan-genome, 3,963 were shared by the seven strains, which accounted for up to 83.1–87.5% of CDS in terms of individual genomes (Figure 4). Therefore, the vast majority of CDS may perform highly conserved functions required by *V. alginolyticus*. As for the non-core genes, the percentages ranged in 8.7–10.2% and 3.8–7.4% for dispensable and specific genes, respectively. Figure 4 shows that the numbers of dispensable genes, shared by two or more strains, dropped sharply; for instance, XWV9 and HNV2 shared the maximal number of 44 dispensable genes, while a maximum of 24 genes were shared by HYV1, HNV2, and FJV2. Thus, the number of shared dispensable genes decreases with more strains involved. In contrast, each strain possessed a high number of specific genes (173–351), which may favor the adaption to respective habitats of the seven strains.

Although no CRISPR was found, the identified GIs, plasmids, and prophages from the seven strains, as well as the number of genes encoded by these MGEs, are listed in Supplementary Tables 1–3. Collectively, very few or none of the core genes were located on plasmids (0–1 genes), prophages (0–3 genes) and GIs (0 gene). Most of the CDSs in MGEs were dispensable or specific, and the majority were annotated as hypothetical proteins and proteins with hitherto unknown functions. The proportions of MGE-specific genes to the total specific genes of individual strains were 13.3%, 14.3%, and 31.6% for SXV3, ZZV2, and XWV9, respectively, and up to 44–45% for ZLV3, FJV2, and HNV2. These results further verify the functions of these mobilome elements in the acquisition of alien genes.

### Phylogenetic Relationship of the 21 *V. alginolyticus* Strains

**Figure 5:**
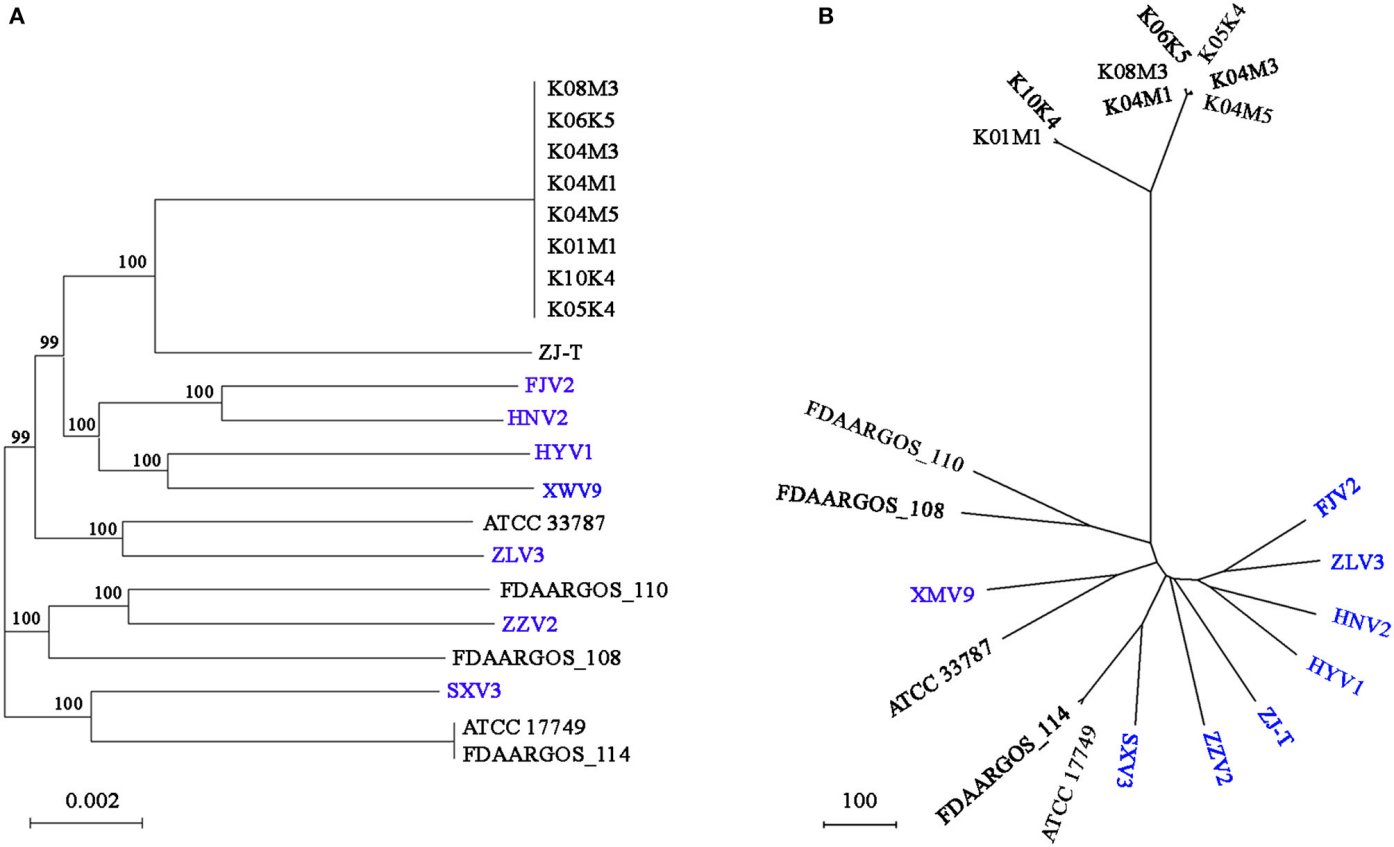
The phylogenomic tree generated based on concatenated alignments of 3854 single-copy core genes among the 21 strains using the ML method. The scale bar indicates the number of substitutions per site **(A)**. The pan-genome phylogeny constructed based on presence/absence of homologous genes with UPGMA method. The scale bar indicates the distance per 100 genes difference **(B)**. ML, maximum likelihood; UPGMA, unweighted pair-group method with arithmetic means; branch labels, bootstrap support values.

Based on the core genome, Figure 5A shows the phylogeny of the 21 *V. alginolyticus* strains. Among the seven strains, FJV2 clustered most closely with HNV2, whereas the distance between isolation sites of these two was farthest regarding pairs of all strains. The eight strains, came from Germany, clustered closely and formed a subclade with ZJ-T obtained from China. Meanwhile, strains FJV2, HNV2, HYV1, and XWV9 grouped into another subclade, then these two subclades formed a branch with ATCC 33787 (from the USA) and ZLV3. Notably, ZZV2 was closely related to FDAARGOS_110 and FDAARGOS_108 derived from England, whereas SXV3 was closely related with FDAARGOS_114 and ATCC 17749 which originated from England and Japan, respectively. Furthermore, the phylogenomic analysis based on the pan-genome also demonstrated this phenomenon of intimate relatedness of strains across diverse nations; for instance, the closer phylogenetic relationship of XWV9 with ATCC 33787, or the robust clustering of SXV3 with FDAARGOS_114 and ATCC 17749 was observed (Figure 5B). These results suggested that no obvious correlation existed between the phylogeny and geographical origin of *V. alginolyticus* strains; furthermore, *V. alginolyticus* strains may not only spread frequently among different coastal provinces in China, but also transfer across nations/continents via trade of aquatic products or human contacts.

### Functional Enrichment of the Pan-Genome

**Figure 6:**
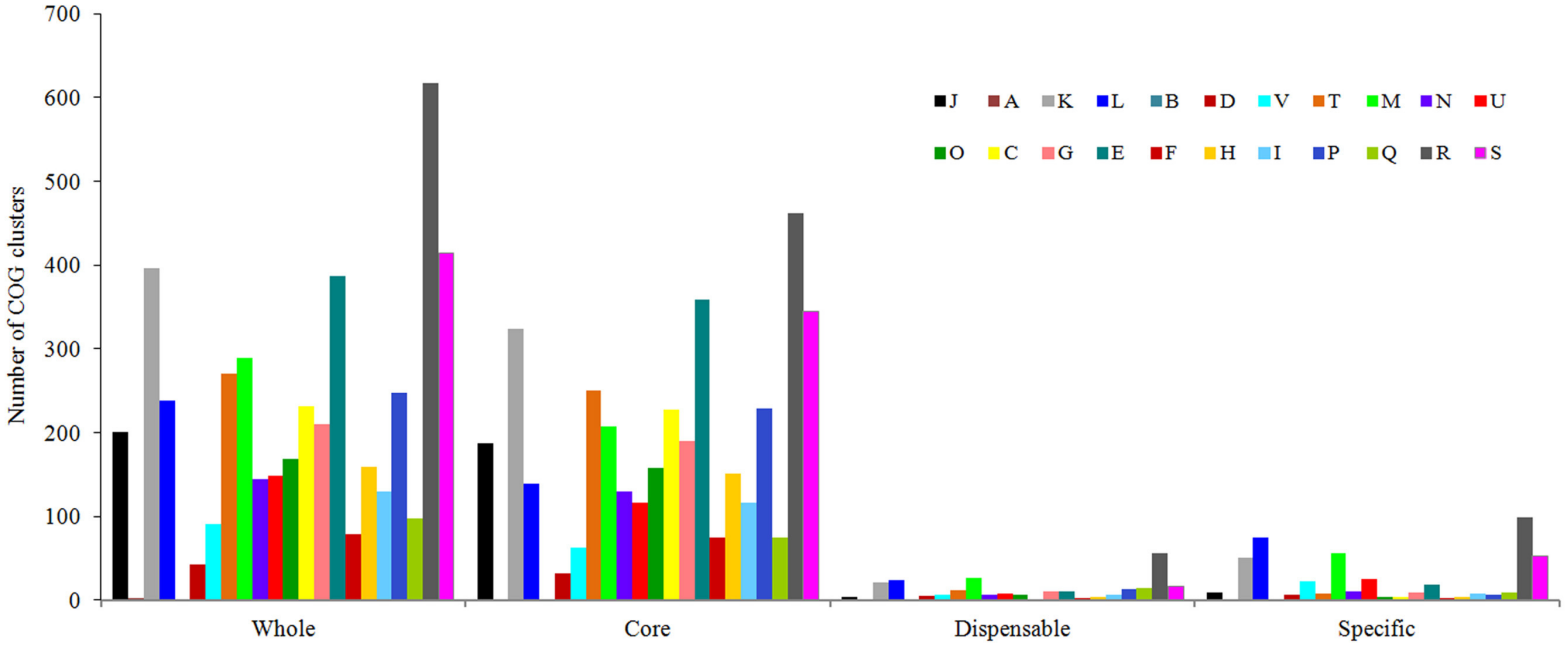
Functional enrichment analysis of COGs in the pan-genome of the seven *V. alginolyticus* strains in terms of the whole-, core-, dispensable- and specific-genes. Designations of functional categories: [J] translation; [A] RNA processing and modification; [K] transcription; [L] replication and repair; [B] chromatin structure and dynamics; [D] cell cycle control and mitosis; [V] defense mechanisms; [T] signal transduction; [M] cell wall/membrane/envelope biogenesis; [N] cell motility; [U] intracellular trafficking and secretion; [O] post-translational modification, protein turnover, chaperones; [C] energy production and conversion; [G] carbohydrate transport and metabolism; [E] amino acid transport and metabolism; [F] nucleotide transport and metabolism; [H] coenzyme transport and metabolism; [I] lipid transport and metabolism; [P] inorganic ion transport and metabolism; [Q] secondary metabolites biosynthesis, transport and catabolism; [R] general function prediction only; [S] function unknown.

As shown in Figure 6, genes belonging to the core genome showed COG function enrichment profiles similar to those of the pan-genome, that is, the relatively abundant COG categories included [K] transcription, [E] amino acid transport and metabolism, [M] cell wall/membrane/envelope biogenesis, [T] signal transduction mechanisms, inorganic ion transport and metabolism, [J] translation, ribosomal structure and biogenesis, and [L] replication, recombination, and repair. In contrast, the enrichment levels of various COGs in accessory genes were relatively quite low, besides [K] transcription, [L] replication, recombination, and repair, and [M] cell wall/membrane/envelope biogenesis, some functions such as [V] defense mechanisms and [U] intracellular trafficking, secretion, and vesicular transport were also enriched to a certain extent, which may facilitate *V. alginolyticus* to overcome adversity. Furthermore, examination of the enriched COGs revealed that ~32.2% of the core genes were poorly characterized as being assigned to [R] general function prediction, [S] unknown function, or even unclassified functions. Meanwhile, 70.5% and 80.9% of dispensable and specific genes, respectively, were also poorly characterized in functions (profile of COG enrichments in Supplementary Table 4). These results indicated that much of the basic biology of *V. alginolyticus* still remains unclear.

### Analysis of the Antibiotic Resistance and Virulence Factors

After resistance prediction using ResFinder online, the seven strains all harbored *blaCARB-42*, which belongs to beta-lactams and may exhibit resistance to ampicillin, piperacillin, and amoxicillin. When annotated in the ARDB database, five identical hits were found, including one *CRP*, one *par*E, and three *ade*Fs with different identities of matching region, which conferred resistance to macrolide, fluoroquinolone, tetracycline, and penicillin. Therefore, the predicted resistance genotypes of the seven strains were highly consistent using either ResFinder or ARDB. After aligning the sequences to the VFDB database with genus *Vibrio* as reference, the numbers of putative virulence genes of individual strains ranged in 151–165 (Supplementary Table 5), of which 146 genes were shared by all strains (Supplementary Table 6), whereas the others were differentially distributed among the seven strains (Table 2). For instance, strains HYV1 and SXV3 had the most similar toxicity profiles with exception of three genes. Specifically, some virulence genes were exclusive in certain strains, such as *msh*B and *wbf*U for ZZV2, *wbf*Y and *wec*C for XWV9, and *cys*C1 for HNV2, all of which may exhibit potential toxic effects via adherence or antiphagocytosis, or act directly as toxins.

**Table 2:** The differed virulence factors among the seven *V. alginolyticus* strains.

**VF class**	**Virulence factors**	**Related genes**	**XWV9**	**HYV1**	**ZLV3**	**SXV3**	**FJV2**	**HNV2**	**ZZV2**
Adherence	MSHA type IV pilus	*msh*B	-	-	-	-	-	-	+
	Type IV pilus	*pil*A	-	+	-	-	-	+	-
Antiphagocytosis	Capsular polysaccharide	*wbf*U	-	-	-	-	-	-	+
		*wbf*Y	+	-	-	-	-	-	-
		*wbj*D*/wec*B	+	-	-	-	-	-	+
		*wec*A	+	+	+	+	+	+	-
		*wec*C	+	-	-	-	-	-	-
		*wzb*	+	+	+	+	+	+	-
	Capsule (*Klebsiella*)	*uge*	-	+	-	-	+	+	-
Iron uptake	ABC transport systems	*viu*C	-	+	-	+	-	-	-
	Vibriobactin	*vib*A	-	+	-	+	-	-	-
		*vib*B	-	+	-	+	-	-	-
		*vib*C	-	+	-	+	-	-	-
		*vib*E	-	+	-	+	-	-	-
	Acinetobactin	*bar*B	+	+	+	+	-	-	-
		*bau*B	+	+	+	+	-	-	-
		*bau*C	+	+	+	+	-	-	-
		*bau*D	+	+	+	+	-	-	-
	Ent siderophore (*Klebsiella*)		-	+	-	+	-	-	-
			-	+	-	+	-	-	-
		*fep*G	-	+	-	+	-	-	-
	Enterobactin transport	*fep*B	-	+	-	+	-	-	-
Toxin	Phytotoxin phaseolotoxin	*cys*C1	-	-	-	-	-	+	-
Immune evasion	Capsule (*Acinetobacter*)		+	+	+	+	+	-	+
	LOS (*Campylobacter*)		-	-	+	+	-	-	-

+ *and* - *indicate the presence or absence of genes, respectively*.

**Figure 7:**
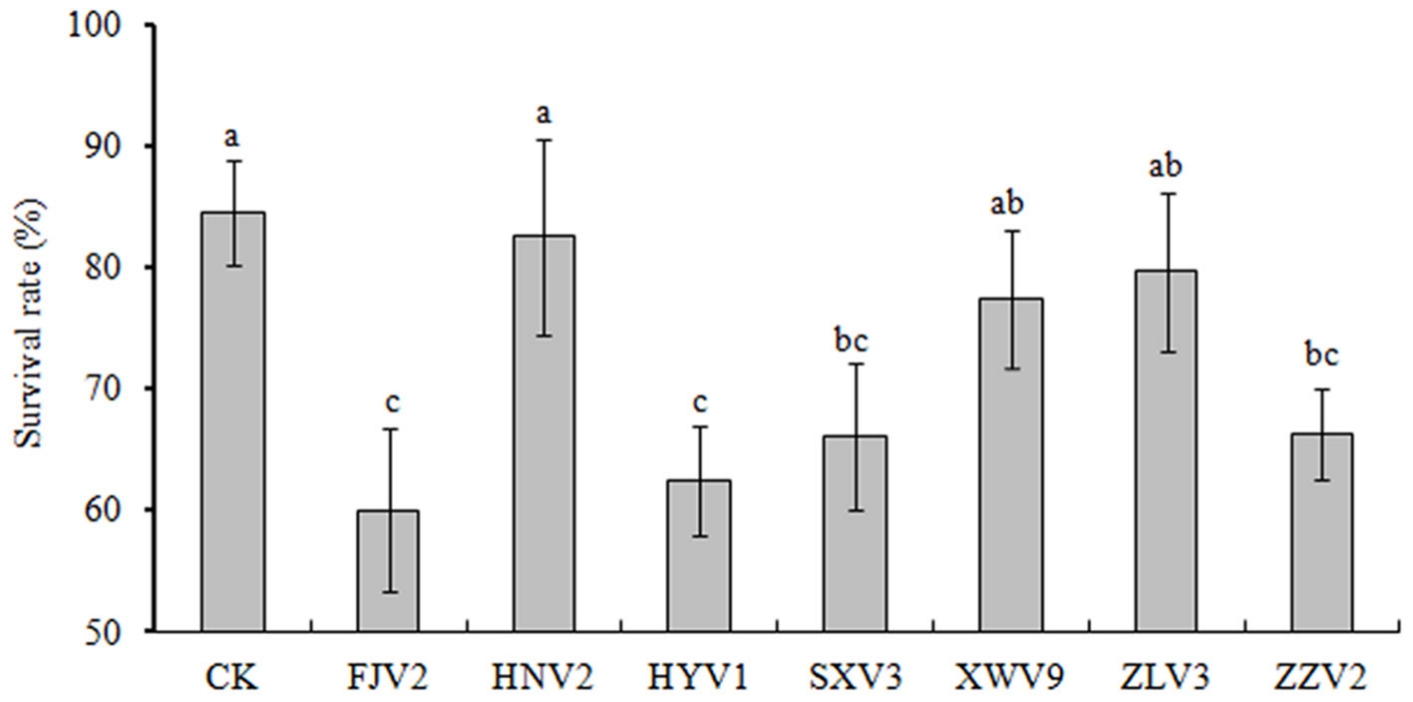
The percentages of survival rate of *L. vannamei* postlarvae after treatment with the seven *V. alginolyticus* strains.

Figure 7 shows the survival of *L. vannamei* postlarvae after treated with the seven strains. Overall, the survival rates in all treatments were lower than that of the control. In particular, treatments with FJV2, HYV1, SXV3, and ZZV2 all significantly reduced the survival rates (*P* < 0.05), and treatment with FJV2 had a minimum survival rate of 60%. In contrast, treatments with HNV2, XWV9, and ZLV3 showed no significant differences in shrimp survival rates when compared to the control. Thus, strains FJV2, HYV1, SXV3, and ZZV2 may be potentially pathogenic to postlarval shrimps, whereas the other three strains may be non-pathogenic.

### Analysis of Genes Related to Chitin Utilization

**Figure 8:**
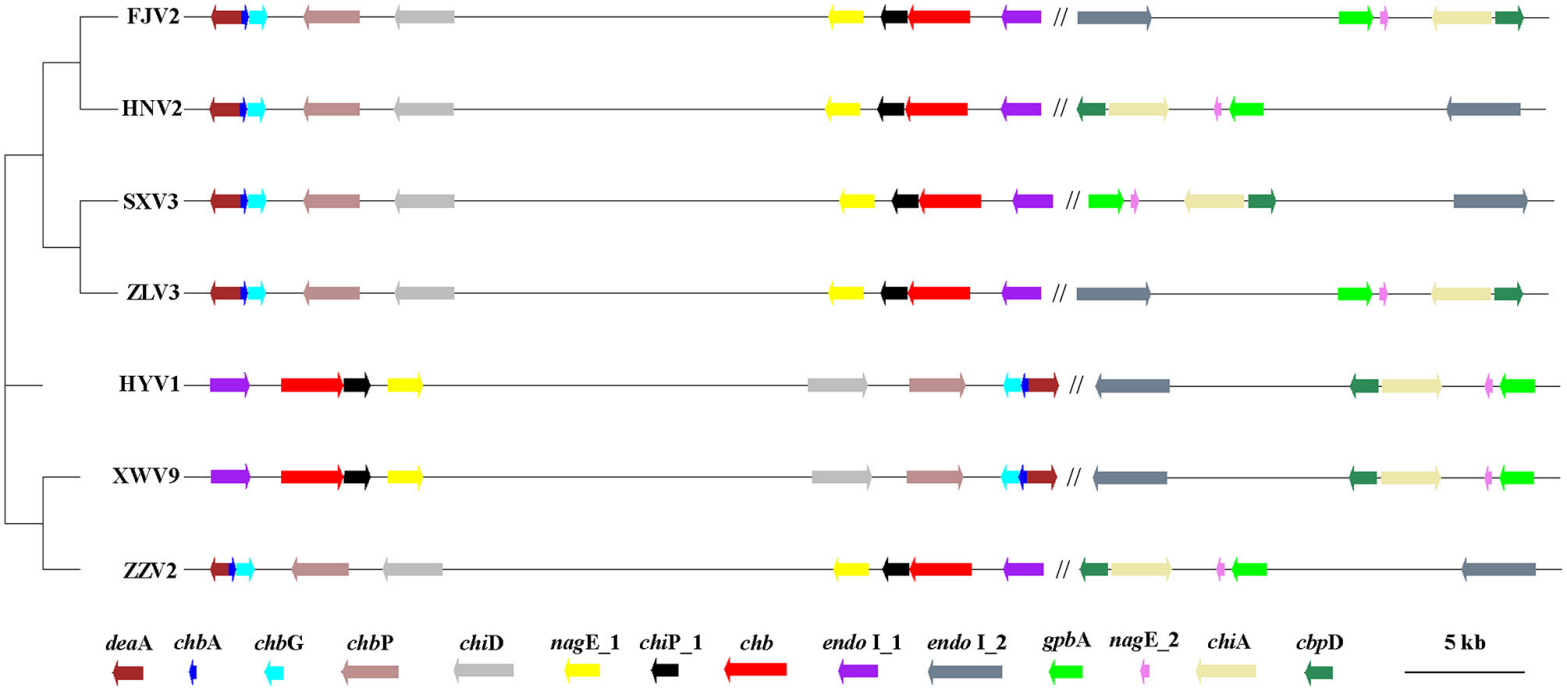
The phylogenetic tree generated using the neighbor-joining method based on the alignment of 14 single-copy genes involved in chitin-degradation pathway of the seven *V. alginolyticus* strains. The first nine genes locate on chromosome I, which being separated by symbol “//” from other five genes on chromosomes II. Arrows to the left and to the right indicate genes encoded on the plus and minus DNA strands, respectively. The scale bar indicates the size of genes, while the distances between genes was reduced by 100 times. Gene annotation: *endo* I_1 and *endo* I_2: Chitodextrinase; *chb, N, N*'-diacetylchitobiase; *chi*P_1: chitoporin; *nag*E_1/*nag*E_2, PTS system *N*-acetylglucosamine-specific EIICBA component; *chi*D, Chitinase D; *chb*P, *N, N'*-diacetylchitobiose phosphorylase; *chb*G, Chitooligosaccharide deacetylase ChbG; *chb*A, PTS system *N, N'*-diacetylchitobiose-specific EIIA component; *dea*A, chitin disaccharide deacetylase; *gbp*A, GlcNAc-binding protein A. *chi*A, chitinase A; *cbp*D, chitin-binding protein CbpD.

A total of 4,620 gene clusters, accounting for 74.31% of the pan-genome, were observed in the seven strains and experienced extensive genetic variation, including base indels and substitution. Among them, 14 core genes, assigned to functions of chitin utilization, were detected on all the seven genomes with a single copy (Figure 8). The annotation, function, size, and gene locus of the 14 genes are listed in Supplementary Table 7. Presumably, these 14 genes may act synergistically to hydrolyze chitin to oligosaccharides or monomer, and the possible degradation process is as follows: extracellular chitinase A (*chi*A) and chitinase D (*chi*D) first degrade chitin into chito-oligosaccharides [(GlcNAc)_n>2_], which being transported into the periplasmic space via specific chitoporin (*chi*P_1*)*. Then, chitodextrinases (*endo* I_1 *and endo* I_2) degrade the oligosaccharides to (GlcNAc)_1,2_ in the periplasm; (GlcNAc)_2_ is transported across the inner membrane by an ABC-type transporter, whereas GlcNAc can be transported into the cytosol and phosphorylated via PTS transporter (*chb*A*, nag*E_1 and *nag*E_2). In the cytosol, (GlcNAc)_2_ is converted into two (GlcNAc-6-P) by an *N, N'*-diacetylchitobiose phosphorylase (*chb*P) and several other mutases or kinases. In addition, chitooligosaccharide deacetylase ChbG (*chb*G) and chitin disaccharide deacetylase (*deaA*) may detach the *N*-acetyl group from the reducing end of (GlcNAc)_2_ with formation of the heterodisaccharide 4-*O*-(*N*-acetyl-β-D-glucosaminyl)-D-glucosamine (GlcNAc-GlcN), where the β-1-4 linkage between the glucosamine residues could be broken by *N, N'*-diacetylchitobiase (*chb*). The chitin-binding proteins CbpD (*cbp*D) and GlcNAc-binding protein A (*gbp*A) may bind to both chitin and its lysis products. The final products are fructose-6-phosphate, acetate, and ammonium, which subsequently enter the metabolic center.

**Figure 9:**
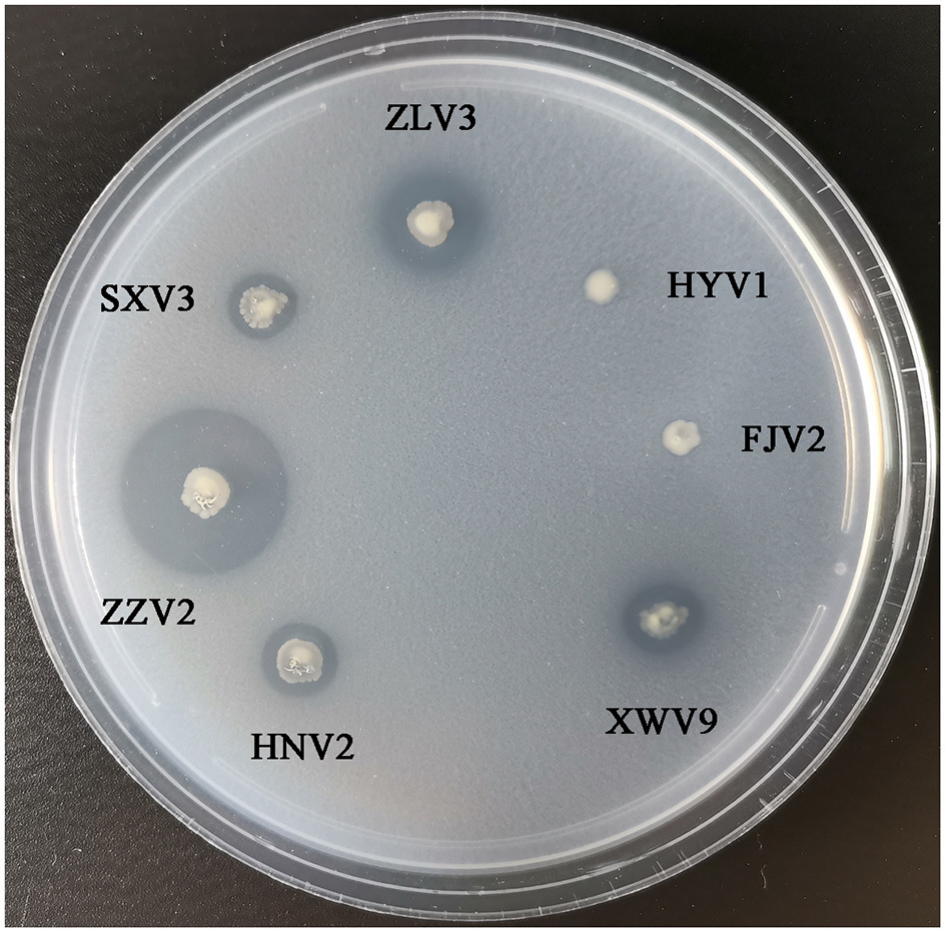
The growth performance of the seven *V. alginolyticus* strains on medium containing chitin as sole source of carbon-nitrogen.

Figure 8 shows the phylogeny of the seven strains based on sequences of 14 genes and the order of their arrangement in the genomes. Notably, phenotypic tests showed that all strains grew well on the medium containing chitin as the sole carbon-nitrogen source (Figure 9), indicating that they can degrade chitin efficiently. However, the sizes of lytic halos differed substantially, and no obvious correlation was noted between the phylogenetic relationships of strains and their levels of chitinolytic ability.

Information on SNP and selection pressure of these genes, including base indels and mutations, are listed in Table 3. Base indels were not detected in most of these genes, except for *dea*A, *chi*A*, endo* I_1, and *nag*E_1, while all the 14 genes had undergone point mutations. Specifically, *dea*A and *nag*E_2 undergone positive selection (*dN/dS* > 1) during evolution, while the remaining 12 genes experienced negative selection owing to the average low level (~0.21) of *dN/dS* not only among seven strains, also among the 21 strains, thus suggesting their pivotal functions.

**Table 3:** The SNP information of 14 core genes involved in chitin-degradation among *V. alginolyticus* strains.

**Gene cluster**	**Among seven strains**				**Among 21 strains**			
	**InDel base**	**Non-synonymous mutation**	**Synonymous mutation**	**dN/dS**	**InDel base**	**Non-synonymous mutation**	**Synonymous mutation**	**dN/dS**
*cbp*D	0	26	86	0.3	0	30	95	0.3
*chb*	0	19	39	0.5	0	32	86	0.4
*chb*G	0	23	41	0.6	0	27	42	0.6
*chb*A	0	1	2	0.5	0	2	6	0.3
*chi*D	0	24	53	0.5	0	33	64	0.5
*dea*A	171	28	13	2.2	171	29	26	1.1
*endo* I_1	7	14	62	0.2	16	42	196	0.2
*chi*P_1	0	2	24	0.1	0	4	28	0.1
*chi*A	9	16	43	0.4	152	20	45	0.4
*endo* I_2	0	10	47	0.2	0	19	61	0.3
*chb*P	0	13	98	0.1	0	26	145	0.2
*gbp*A	0	15	19	0.8	0	20	25	0.8
*nag*E_1	0	0	14	0.0	14	3	36	0.1
*nag*E_2	0	5	2	2.5	0	7	5	1.4

## Discussion

In the present study, each of the seven strains possessed two chromosomes and 0–2 plasmids, similar to those reported in other *V. alginolyticus* strains (Thompson et al., 2009; Lin et al., 2018; Chibani et al., 2020). Genomic comparison demonstrated that although the chromosome I of *V. alginolyticus* did not differ greatly in size (3.27–3.40 Mb) relative to those of *V. cholerae* and *V. parahaemolyticus* (3.0 and 3.3 Mb, respectively), the size (1.81–1.89 Mb) of chromosome II was closer to that of *V. parahaemolyticus* (1.9 Mb) than that of *V. cholerae* (1.1 Mb) (Tagomori et al., 2002; Makino et al., 2003). These results further verified that chromosomes of vibrios play different roles. Specifically, the relatively stable structure of chromosome I may be associated with conserved functions such as those for growth and metabolism, whereas the high variability in chromosome II is attributed to bacterial niche adaptation (Makino et al., 2003; Thompson et al., 2009). As for plasmids, the differences in size varied greatly not only among the seven strains in this study, also among the Kiel-alginolyticus strains (Chibani et al., 2020) and *V. alginolyticus* ATCC 33787 (Wang et al., 2016). These results indicate that transfer of plasmids may occur less within strains and more among species, e.g., the 93% sequence similarity of a virulence-associated plasmid from a shrimp pathogen *V. nigripulchritudo* to a plasmid from the coral pathogen *V. shilonii* (Reynaud et al., 2008). Furthermore, the Korean *V. parahaemolyticus* strains harbored the Asian-type AHPND plasmid, which was almost identical to the AHPND-associated *V. owensii* plasmid pVOWZ2 previously identified in China (Han et al., 2020). Therefore, plasmids may play a crucial role in the transfer of genes required by vibrios to infect marine animals.

An open bacterial pan-genome shows that new genes are continuously added to the gene pool of the species once a new strain is sequenced (Tettelin et al., 2005). In this study, the pan-genome size of 6,217 among the seven strains compared to 9,241 among 21 strains indicated that greater niche diversity of *V. alginolyticus* will require a larger pan-genome, given the changing environments where vibrios are distributed. Thompson et al. (2009) reported that *Vibrio* had a vast gene repertoire with a pan-genome of >26,500 genes (43 strains from 17 species), which was 50 times larger than that of the *Vibrio* core genome; nevertheless, the ratio of pan-genome (6,923) to core genome of 18 *V. cholerae* strains decreased substantially to approximately 4.6. Therefore, the relatively smaller pan-genomes of *V. cholerae* and *V. alginolyticus* when compared to those of *Vibrio* genus might reflect a specific set of genes required to occupy the species-specific niche. Remarkably, each additional sequenced genome contributed ≥ 161 and ≥ 256 new genes to the *V. alginolyticus* pan-genome of the 21 and seven strains, respectively. Similarly, the open pan-genome of *Streptococcus agalactiae* constantly identify unique genes after sequencing hundreds of genomes (Tettelin et al., 2008). The open pan-genome of *Escherichia coli* indicated that sequencing could result in identification of ca. 300 novel genes per genome (Rasko et al., 2008). Therefore, *V. alginolyticus*, with an open pan-genome, is evolving continuously through gene acquisition to achieve high genome plasticity.

Regarding the core genome, the very close numbers of 3,963 and 3,894 accounted for 64% and 42% of the pan-genome for the seven and 21 strains, respectively. Likewise, previous studies have reported that the percentages of conserved core genes were 46% and 50% for 17 *Streptococcus pneumoniae* and 13 *Haemophilus influenzae* strains, respectively (Hiller et al., 2007; Hogg et al., 2007). The *E. coli* pan-genome contains a reservoir of more than 13,000 genes, approximately 17% of which constitute the core genome (Rasko et al., 2008). Tettelin et al. (2005) reported that the core genome of eight *S*. *agalactiae* strains may reach a minimum of 1,806 genes and will remain relatively constant as more genomes being added. Therefore, the size of the core genome of several bacteria was stable although with varied proportions, which depending on the number of strains analyzed. Considering the high percentage of core genes (82%) relative to the average genome size of 4,843 of the seven *V. alginolyticus* strains, the vast majority of coding genes may be functionally conserved across diverse strains.

Various MGEs, including plasmids, prophages, and GIs identified in the seven strains, encoded mainly the dispensable and specific genes, which were similar to those reported for other bacterial species (Rasko et al., 2008; Hazen et al., 2010; Lin et al., 2018; Deng et al., 2019). The vast majority of these MGEs-encoded genes, though with unclear functions, may enhance the genome plasticity of *V. alginolyticus* to compete with other organisms (Lin et al., 2018). In particular, the proportions of MGEs-specific genes of strains ZLV3, FJV2, and HNV2 accounted for up to 44–45% of total strain-specific genes, demonstrating that the main function of MGEs is to facilitate HGT events, which ultimately results in niche expansion and evolution of vibrios (Thompson et al., 2009; Lin et al., 2018; Chibani et al., 2020). Among the few genes being annotated, some of those were closely related to replication, integration and excision of MGEs, some were virulence-associated genes, e.g., there were four *vir*B genes encoding Type IV secretion system (T4SS) proteins in pL33_1 and eight *vir*B T4SS genes in pL33_2 of strain FJV2, gene *vir*B was also found in plasmid pL40 of strain HYV1, another two virulence genes, *apx*IB_1 and *cva*A, encoding toxin RTX-I translocation ATP-binding protein and colicin V secretion protein, located in GIs of FJV2, all these indicated that frequent transfer of virulent factors among diverse strains occurred via MGEs as previously reported (Paixão et al., 2016; Costa et al., 2021). Surprisingly, gene *xer*C, enconding tyrosine recombinase, was found to distribute widely in various MGEs in this study, which including prophages of strains SXV3 and ZZV2, GIs of FJV2, SXV3, ZZV2, and ZLV3, plasmids of XWV9 and HNV2, so this gene may participate in horizontal dissemination of resistant genes among strains as suggested by Lin et al. (2020). Therefore, these MGEs may play a significant role in the emergence of toxigenic or resistant strains of *V. alginolyticus* from environmental non-toxigenic or antibiotic-sensitive populations (Boucher et al., 2011).

The number of putative virulence factors of seven isolates ranged from 151 to 165, which was slightly higher than those (149–150) found in nine strains of the Kiel-*alginolyticus* ecotype (Chibani et al., 2020). Of the 146 virulence factors shared by the seven strains, four genes (*wza, vop*D, *vop*B, and *hcp*) had also been proven to exist in 30 environmental strains of *V. alginolyticus* detected using PCR (Hernández-Robles et al., 2016). The gene *tlh*, encoding thermolabile hemolysin, was observed in all strains in this study, but it was detected in only six of the 62 *V. alginolyticus* strains that were previously isolated from the coastal mariculture system in Guangdong province, China (Xie et al., 2005). Therefore, increased frequency in detection of *tlh* with time might indicate a more frequent gene transfer of this gene among diverse *V. alginolyticus* strains since rearing water is usually drawn from coastal seawater. Ren et al. (2013) reported that seven virulence genes, namely *ctx*B, *zot, tag*A, *stn, sto, tdh*, and *trh*, were detected only in certain pathogenic *V. alginolyticus* isolates, but not in non-pathogenic strains. These genes were not detected in the strains isolated in this study, although four strains were potentially opportunistic pathogens. These results suggest that divergent levels of potential toxicity have evolved in *V. alginolyticus* strains through the selection of virulence factors.

Some strains possessed unique virulence genes, such as *msh*B and *wbf*U involved in adherence and antiphagocytosis respectively were found only in ZZV2, thus the exact toxic effects of these two genes warrant further investigation as shrimp postlarvae presented significantly lower survival rates after treated with strain ZZV2. Additionally, *wbf*Y and *wec*C, both related to antiphagocytosis, were unique to XWV9, whereas gene *cys*C1, encoding a specific phytotoxin phaseolotoxin, was detected only in HNV2. However, these three genes did not exhibit toxic effects on shrimp. Overall, no obvious correlation was noted between the existence of specific virulent genes and the level of strain toxicity. For instance, strains HNV2 and FJV2 exhibited maximal and minimal postlarval survival of 83% and 60%, respectively, whereas only three virulence factors differed between them, the genes *pil*A and *cys*C1 were only found in HNV2, while another gene associated with capsule formation only emerged in FJV2, however, this gene was not necessarily toxic as it also emerged in non-pathogenic strains XWV9 and ZLV3. Likewise, Xie et al. (2005) demonstrated no positive correlation between virulence phenotype and genotype in *V. alginolyticus*, as some strains were pathogenic to fish, although they did not possess any detectable virulence genes, contrarily, others were non-pathogenic despite the presence of virulence genes. Busschaert et al. (2015) suggested that virulence is multifactorial because there was no correlation between the presence of SNPs/indels in virulent factors of 15 *V. anguillarium* strains and their toxicity toward *Dicentrarchus labrax* larvae. Nevertheless, as members of the Harveyi clade, these *V. alginolyticus* strains may serve as a large reservoir of putative virulence genes to transform a non-pathogenic strain into a pathogenic strain as suggested by Faruque and Nair (2002) and Ruwandeepika et al. (2010). Further study is needed to ascertain the real virulent factors of *V. alginolyticus* to shrimp individuals.

Considering the variety of virulence genes carried by each strain in normal shrimp larviculture water, the relative abundance of *V. alginolyticus* strains should be monitored to avoid vibriosis outbreaks. Remarkably, besides the robust phylogenetic relationship of strains SXV3 and ATCC 17749, there were only three virulent factors differed between them, i.e., *msh*B and *pil*A possessed only by ATCC 17749, while a putative gene of LOS (*Campylobacter*) associated with immune evasion harbored by SXV3. Furthermore, ATCC 17749 may be pathogenic since it was obtained from the spoiled horse mackerel causing food poisoning (Liu et al., 2015), while SXV3 was potentially pathogenic to shrimp postlarvae in this study. This high similarity in toxicity profiles might indicate a cross-nation transfer of *V. alginolyticus*. Therefore, the wide transmission of potentially pathogenic vibrios during seafood trade among different nations should receive great attention.

Chitin degradation is a common feature for some marine vibrios to acquire nutrients and is achieved by a complex pathway that includes multiple chitinases, binding proteins, and related transport factors (Meibom et al., 2004; Markov et al., 2015). In this study, a total of 14 genes involved in the chitin degradation pathway, which may perform synergistically to hydrolyze chitin, were retrieved from the seven *V. alginolyticus* genomes. In terms of gene arrangement, nine genes located on chromosome I were more regular in seven strains than those on chromosome II, indicating that genes on small chromosome may be more likely subjected to recombination or HGT events during evolution, as described by Nei and Rooney (2005). Except for *dea*A and *nag*E_2, synonymous mutations were noted more than non-synonymous mutations for other genes. The 12 genes had been subjected to significant purifying selection (*dN*/*dS* <1), wherein seven genes, including *nag*E_1, *endo* I_1, *endo* I_2, *chi*P_1, *cbp*D, *chb*P, and *chi*A, presented *dN*/*dS* lower than 0.5. The lower pressure of negative selection on these seven genes indicated that they may play more significant roles in the chitin-degradation process in vibrios. Among these genes, *chi*A is considered to be crucial in the chitin degradation pathway of vibrios. Lin et al. (2018) deduced that *chi*A, which evolved along with *Vibrio*, had undergone remarkable negative selection to conserve its ancestral state. In contrast, genes *dea*A and *nag*E_2 had experienced obvious positive selection (*dN*/*dS* >1), which may be attributed to the existence of other genes with similar functions, such as *chb*G and *nag*E_1. Therefore, these genes could endure multiple genetic events, including non-synonymous mutations.

In this study, all the 14 genes, associated with chitin degradation, exclusively belonged to the core genome with a single copy, which indicates the importance of chitin utilization in vibrios (Hunt et al., 2008; Markov et al., 2015; Lin et al., 2018). Furthermore, the ability of these strains to grow well on substrates using chitin as the sole carbon-nitrogen source confirmed that they produce chitinases to decompose chitin. Shrimp rearing systems generally contain a tremendous amount of shells/carapaces molted from live shrimps or from dead individuals. The presence of these chitin-rich exoskeletons in shrimp larviculture water may serve as a good nutrient medium for diverse vibrios. In particular, some opportunistic pathogenic vibrios, such as strains FJV2, HYV1, SXV3, and ZZV2, may proliferate rapidly to infect shrimp, ultimately resulting in vibriosis. Therefore, the dominant distribution of *V. alginolyticus* in shrimp larviculture system may be closely associated with pervasive chitin and its derivatives; therefore, timely removal of diverse chitin-rich materials should be a key strategy to avoid frequent vibriosis.

Remarkably, some of the chitin-degradation genes have been reported as virulence factors in some vibrios, for example, gene *chi*A is typical toxic for the Harveyi clade (Defoirdt et al., 2010; Ruwandeepika et al., 2010) and gene *gpb*A is a key factor for *V. cholera* to colonize the chitinous exoskeleton of zooplankton and intestinal epithelium of human (Wong et al., 2012). Thus, the potential toxic effects of the 14 genes related to chitin utilization in *V. alginolyticus* warrant investigation since shrimps are chitin-rich in their exoskeleton and peritrophic membrane of intestine. Furthermore, considering the differed pathogenic phenotypes between diverse strains of *V. alginolyticus*, further studies are required to ascertain the impacts of non-pathogenic vibrios on shrimp performance regarding chitin-degradation in intestine.

In conclusion, *V. alginolyticus* has an open pan-genome with increasing gene content, although its core genome is fairly stable. Most MGE-related genes, such as those encoded on plasmids, prophages, and GIs, are dispensable or specific genes with hitherto unknown functions. Meanwhile, seven *V. alginolyticus* strains presented identical antibiotic resistance profiles and highly similar virulence factors, irrespective of their differed toxicity effects on *L. vannamei* postlarvae. In addition, 12 out of a total of 14 genes involved in the chitin degradation pathway had undergone significant purifying selection (*dN*/*dS* < 1), and all seven strains could utilize chitin as sole carbon-nitrogen source. Therefore, the persistence of *V. alginolyticus* in various aquatic environments may be attributed not only to the high genomic plasticity via continuous acquisition of alien genes by diverse MGEs, but also to the crucial role conferred by conserved core genomes, such as chitin utilization. In particular, the dominance of vibrios in shrimp culture environments may be closely associated with the existence of rich shells or carapaces. To avoid the frequent occurrence of vibriosis in *Penaeus* shrimp, timely removal of diverse chitin-rich materials may be crucial to prevent the rapid proliferation of diverse opportunistic pathogenic *V. alginolyticus* strains. Furthermore, the potential transcontinental transmission of vibrios due to aquatic trade should also receive great attention.

## Data Availability Statement

The datasets presented in this study can be found in online repositories. The names of the repository/repositories and accession number(s) can be found in the article/Supplementary Material.

## Author Contributions

CW and MX conceptualized, designed the experiments, analyzed the data, and wrote the manuscript. CW, XH, MX, JX, RH, GL, HL, and JL performed the experiments. All authors contributed to the article and approved the submitted version.

## Funding

This study was supported by the National Natural Science Foundation of China (Nos. 32072995 and 31372536), the Science and Technology Project of Zhanjiang City (No. 2019A04005), and the Special Fund for Innovation and Strengthening of Guangdong Ocean University (No. 230419095).

## Conflict of Interest

The authors declare that the research was conducted in the absence of any commercial or financial relationships that could be construed as a potential conflict of interest.

## Publisher's Note

All claims expressed in this article are solely those of the authors and do not necessarily represent those of their affiliated organizations, or those of the publisher, the editors and the reviewers. Any product that may be evaluated in this article, or claim that may be made by its manufacturer, is not guaranteed or endorsed by the publisher.

## Supplementary Material

The Supplementary Material for this article can be found online at:
