# Supplementary material for: Comparative Genomic Analysis of Seven Vibrio alginolyticus Strains Isolated From Shrimp Larviculture Water With Emphasis on Chitin Utilization
Source: Front Microbiol. 2022 Jul 26;13:925747. doi: 10.3389/fmicb.2022.925747 (PMC9364117; doi:10.3389/fmicb.2022.925747)
Supplement: Supplementary file 1 [file Table_1.docx]

**Supplementary Table 1. The information of genomic island and numbers of their encoded various gene types of pan-genome identified in *V. alginolyticus* strains**

| Strain | Genome position | Region length (bp) | No. of total genes | No. of core genes | No. of dispensable genes | No. of specific genes | No. of hypothetical proteins |
| --- | --- | --- | --- | --- | --- | --- | --- |
| XWV9 | ChrⅠ: 383450-393650 | 10201 | 10 | 0 | 10 | 0 | 6 |
|  | ChrⅠ: 1392632-1442333 | 49702 | 67 | 0 | 47 | 20 | 57 |
| ZLV3 | ChrⅠ: 465381-485019 | 19639 | 19 | 0 | 9 | 10 | 17 |
|  | ChrⅡ: 897192-908482 | 11291 | 12 | 0 | 1 | 11 | 7 |
| SXV3 | ChrⅠ: 31532-46240 | 14709 | 16 | 0 | 6 | 10 | 14 |
|  | ChrⅠ: 2922615-2940566 | 17952 | 21 | 0 | 1 | 20 | 16 |
| FJV2 | ChrⅠ: 373169-385016 | 11848 | 13 | 0 | 1 | 12 | 10 |
|  | ChrⅠ: 2845579-2866807 | 21229 | 25 | 0 | 10 | 15 | 19 |
| HNV2 | ChrⅡ: 1116530-1146119 | 29590 | 32 | 0 | 5 | 27 | 26 |
| ZZV2 | ChrⅠ: 2921331-2939360 | 18030 | 19 | 0 | 1 | 18 | 16 |

**Supplementary Table 2. The information of plasmid and numbers of their encoded various gene types of pan-genome identified in *V. alginolyticus* strains**

| Strain | Genome position | Region length (bp) | No. of total genes | No. of core genes | No. of dispensable genes | No. of specific genes | No. of hypothetical proteins |
| --- | --- | --- | --- | --- | --- | --- | --- |
| XWV9 | pL93 (circular) | 93,384 | 115 | 1 | 35 | 79 | 102 |
| HYV1 | pL40 (linear) | 40,196 | 49 | 2 | 4 | 43 | 35 |
| FJV2 | pL33_1 (circular) | 33,998 | 41 | 1 | 0 | 40 | 26 |
|  | pL33_2 (circular) | 33,975 | 46 | 1 | 0 | 45 | 29 |
| ZZV2 | pL90 (circular) | 90,854 | 111 | 2 | 37 | 72 | 96 |

**Supplementary Table3. The information of prophage and numbers of their encoded various gene types of pan-genome identified in *V. alginolyticus* strains**

| Strain | Genome position | Region length (kb) | No. of total genes | No. of core genes | No. of dispensable genes | No. of specific genes | No. of hypothetical proteins |
| --- | --- | --- | --- | --- | --- | --- | --- |
| XWV9 | ChrⅠ: 2282685-2316466 | 33.7 | 43 | 3 | 28 | 12 | 35 |
| HYV1 | ChrⅠ: 1337141-1378102 | 40.9 | 42 | 0 | 32 | 10 | 10 |
|  | ChrⅠ: 1668088-1676401 | 8.3 | 10 | 0 | 1 | 9/ | 2 |
|  | ChrⅡ: 682881-701308 | 18.4 | 31 | 0 | 15 | 16 | 8 |
| ZLV3 | ChrⅡ: 643592-653031 | 9.4 | 13 | 0 | 11 | 2 | 6 |
| SXV3 | ChrⅠ: 1012070-1048477 | 36.4 | 43 | 0 | 15 | 28 | 23 |
|  | ChrⅠ: 1546185-1583386 | 37.2 | 57 | 2 | 0 | 55 | 37 |
| HNV2 | ChrⅠ: 1364970-1396697 | 31.7 | 21 | 0 | 16 | 5 | 9 |
| ZZV2 | ChrⅠ: 1859795-1889693 | 29.8 | 23 | 0 | 23 | 0 | 8 |
